# Dietary potassium supplementation improves vascular structure and ameliorates the damage caused by cerebral ischemia in normotensive rats

**DOI:** 10.1186/1743-7075-5-3

**Published:** 2008-01-31

**Authors:** Christine' S Rigsby, David M Pollock, Anne M Dorrance

**Affiliations:** 1 Department of Physiology, Medical College of Georgia, 1120 15^th ^Street, Augusta, GA 30912, USA; 2 Vascular Biology Center, Medical College of Georgia, 1120 15^th ^ Street, Augusta, GA 30912, USA; 3 Department of Pharmacology and Toxicology, Michigan State University, East Lansing, MI 48824, USA

## Abstract

**Background:**

Dietary potassium supplementation in hypertensive rats is cardioprotective. This protection includes a blood pressure independent reduction in the amount of damage caused by cerebral ischemia. Therefore, we hypothesized that dietary potassium supplementation would improve the outcome of ischemic stroke by improving cerebral vessel structure in normotensive rats.

**Methods:**

Wistar Kyoto (WKY) rats were fed a high (HK) or low potassium (LK) diet for six weeks from six weeks of age. At the end of treatment, cerebral ischemia was induced by middle cerebral artery (MCA) occlusion and the resultant infarct was quantified and expressed as a percentage of the hemisphere infarcted (%HI). MCA structure was assessed in an additional group of rats using a pressurized arteriograph.

**Results:**

The cerebral infarct was significantly smaller in rats fed the HK diet, compared to rats fed the LK diet (21 ± 5.4 vs 33.5 ± 4.8 %HI HK vs LK p < 0.05). Vessel structure was improved in WKY rats fed the HK diet as indicated by an increase in the MCA lumen (298 ± 6.3 vs 276 ± 3.9 μm HK vs LK p < 0.05) and outer diameters (322 ± 7.6 vs 305 ± 4.8 μm HK vs LK p < 0.05). Wall thickness and area were unchanged, suggesting an outward euthrophic remodelling process. The HK diet had no effect on body weight or telemetry blood pressure.

**Conclusion:**

These studies are the first to show a beneficial effect of dietary potassium in rats with normal blood pressure.

## Background

Epidemiological studies show that potassium intake correlates inversely with the incidence of cerebrovascular events [1-4]. It has been clear for some time that a high potassium diet reduces the number of hemorrhagic strokes in stroke prone spontaneously hypertensive rats (SHRSP) [5,6] and reduces the risk of stroke in humans [1-3]. We have also previously shown that administration of spironolactone, a potassium sparing drug, reduces the ischemic cerebral infarct size in SHRSP [7]. We have recently shown that dietary potassium supplementation also reduces the size of ischemic cerebral infarcts in SHRSP and improves the structure of the MCA [8]. Interestingly, dietary potassium supplementation did not alter blood pressure in the SHRSP fed a normal sodium diet. This presents the possibility that the effects of potassium supplementation are blood pressure independent and may also occur in normotensive rats.

Sixty to eighty percent of ischemic strokes can be attributed to hypertension [9]. However, there remains a significant population that suffers from an ischemic stroke while their blood pressure is normal. It would therefore seem prudent to assess if therapies that have been shown to be beneficial in hypertensive rats can also reduce the ischemic damage in rats with normal blood pressure. Therefore we designed experiments to test the effects of a HK diet in normotensive WKY rats.

## Methods

### Animals

Male WKY rats were purchased from Harlan (Indianapolis IN). Rats were maintained on a 12-hour light dark cycle, housed two per cage and allowed access to food and water *ad libitum*. Rats were fed either a low potassium (LK) or a high potassium (HK) chow containing 0.79% and 2.11% potassium respectively (Zeigler Brothers: Gardners PA) from 6 to 12 weeks of age. Both the HK and LK diets contained 0.16% NaCl. These studies complied with the protocols for animal use outlined by the American Physiological Society and were approved by the Institutional Animal Care and Use Committee.

### MCA occlusion

Rats were anesthetized with sodium pentobarbital (50 mg/kg IP) and body temperature was maintained at 37°C during anaesthesia. The MCA was permanently occluded using the intralumenal thread occlusion technique [10]. Briefly, the common carotid artery was exposed by a midline incision and the branches of the external carotid artery were cauterized. A 3-0 monofilament thread with a rounded tip was introduced into the carotid artery and advanced cranially into the internal carotid artery over a distance of 19 mm, measured from the bifurcation of the common carotid artery. The thread was left in place and the rats were allowed to recover. Blood flow to the region surrounding the MCA was measured using a laser Doppler flow probe to confirm MCA occlusion. Post-occlusion (24 hours), the rats were anesthetized and decapitated and the brains carefully removed. The area of the infarction was quantified using 2,3,5-triphenyltetrazolium (TTC) staining as described previously. The area of the brain that stained pink in response to TTC was deemed to be viable tissue and the white area was classified as tissue damaged by ischemia [7].

### MCA structure

MCAs were isolated and placed in cold physiological salt solution (PSS) (in mM: 141.9 NaCl, 4.7 KCl, 1.7 MgSO_4_, 0.5 EDTA, 2.8 CaCl_2_, 10.0 HEPES, 1.2 KH_2_PO_4_, and 5.0 glucose). The first branch-free segment of the MCA most proximal to the Circle of Willis was mounted on two glass micropipettes in a small vessel arteriograph (Living Systems Instrumentation, Burlington, VT). Vessels were bathed with warm oxygenated PSS and the intralumenal pressure was set at 75 mmHg before the vessels were allowed to equilibrate for 30 minutes. Vessels that did not hold pressure were discarded (vessels from 3 control rats were discarded for this reason). For the analysis of vessel structure, the vessels were bathed in calcium free PSS containing 2 mM EGTA and the intralumenal pressure was increased from 0 to 180 mmHg in 20 mmHg increments. Videomicroscopy was used to measure lumen diameter, external diameter and wall thickness at each pressure after a 5-minute equilibration. The wall/lumen ratio, circumferential wall stress, wall strain and distensibility were calculated using the method of Baumbach and Hadju (13). The vessel growth and remodelling indices were calculated for vessels at an intralumenal pressure of 80 mmHg using the formula described by Heagerty *et al *[11]. The elastic modulus (β-coefficient) was calculated from the stress/strain curves for the individual vessels, these curves were fitted to an exponential model (y = ae^βx^) where β is the slope of the curve: the higher the β-coefficient the stiffer the vessel.

### Blood pressure and plasma electrolytes

Blood pressure was continuously monitored by telemetry [12] (Data Sciences, Inc.; 20). Telemetry probes were placed in the abdominal aorta of five-week old rats. Baseline blood pressure was measured for 3 days before the animals were placed on the specialized diets at six week of age. Plasma sodium and potassium was measured using the Synchron EL-ISE electrolyte system (Beckman Instruments).

### Chemicals

All general laboratory chemicals were purchased from Sigma (St. Louis MO).

### Statistics

All results are represented as a mean ± standard error of the mean. Cerebral vascular structure data and blood pressures were analyzed by two-way repeated measures ANOVA with a Bonferroni post-test. Cerebral infarct size, plasma electrolytes, body weights and β-coefficients were compared using Student's t-test. A p-value ≤ 0.05 was considered significant.

## Results

### Blood pressure, plasma electrolytes and body weights

**Figure 1:**
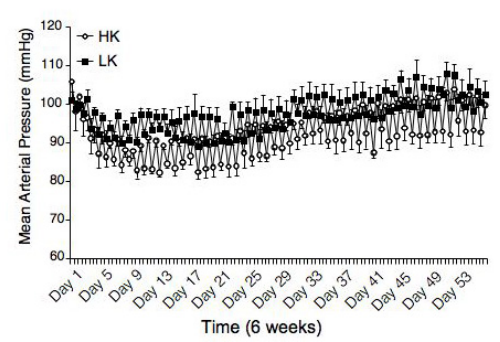
Dietary potassium supplementation did not alter blood pressure in the WKY rats. Blood pressure was measured by telemetry and the average day and night blood pressures are shown (n = 3 in each group).

Dietary potassium supplementation had no effect on the mean arterial blood pressure in the WKY rats (Figure 1). The rats from the two treatment groups had similar body weights (274 ± 4.5 vs 265 ± 5.4 grams HK vs LK). The plasma sodium levels were also similar between the two groups (142 ± 1.8 vs 144 ± 1.7 HK vs LK), there was a trend toward an increase in the plasma potassium levels in the rats fed the HK diet (5.2 ± 0.2 vs 4.9 ± 0.2 HK vs LK p = 0.25).

### Cerebral infract size

**Figure 2:**
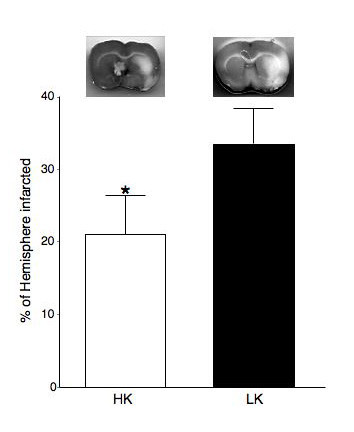
Dietary potassium supplementation reduces the damage caused by cerebral ischemia in WKY rats. Cerebral ischemia was induced by MCA occlusion using the intralumenal suture occlusion technique and the size of the ischemic infarct was measured twenty-four hours later. The upper panel shows representative brain slices from WKY rats fed the HK and LK diets. The light area is tissue damaged by ischemia and the dark area is viable tissue. The lower panel shows the cerebral infarct size expressed as a percentage of the hemisphere damaged by ischemia (n = 6 in both groups, p < 0.05).

Cerebral ischemia was induced by MCA occlusion. After 24 hours of ischemia the cerebral infarct was significantly smaller in rats fed the HK diet, compared to rats fed the LK diet (21 ± 5.4 vs 33.5 ± 4.8 %HI HK vs LK p < 0.05) (Figure 2). The percentage drop in blood flow, measured by laser Doppler was the same in both groups of rats (data not shown) indicating that the MCA was occluded to the same extent in both groups and therefore the difference in infarct size is due to differences in the ability of the vessels to respond to ischemia and not a physical difference in the way the MCA was occluded.

### Passive vessel dynamics

**Figure 3:**
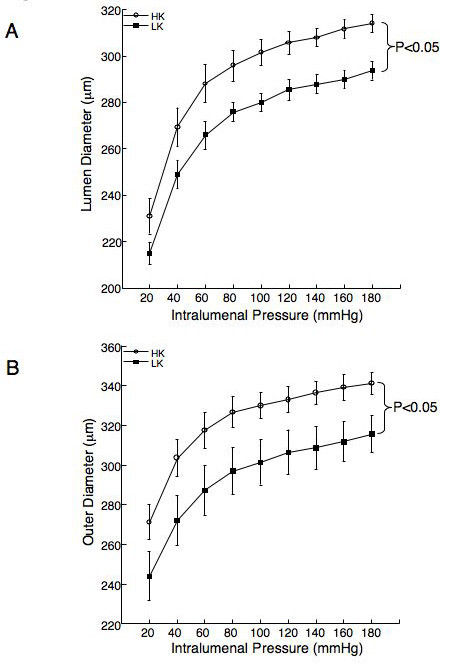
Dietary potassium supplementation increases the inner (A) and outer diameter (B) of the middle cerebral artery. Arteries were mounted in a small vessel arteriograph and the inner and outer diameters of the vessels were measured over a range of intralumenal pressures (20–180 mmHg) under zero flow and calcium free conditions. Results were compared by a two way repeated measures ANOVA (p < 0.05) n = 5 for the HK group and 6 for the LK group).

**Figure 4:**
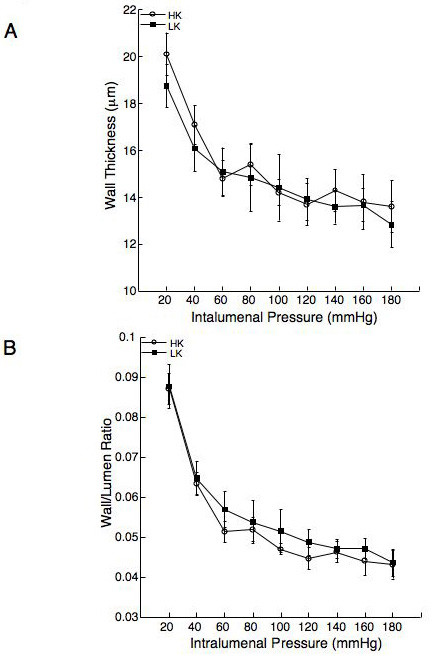
Dietary potassium supplementation has no effect on the wall thickness (A) or the wall/lumen ratio in the WYK rats. Arteries were mounted in a small vessel arteriograph and the wall thickness and lumen diameters of the vessels were measured over a range of intralumenal pressures (20–180 mmHg) under zero flow and calcium free conditions and the wall/lumen ratio was calculated. Results were compared by a two way repeated measures ANOVA (NS) n = 5 for the HK group and 6 for the LK group.

**Figure 5:**
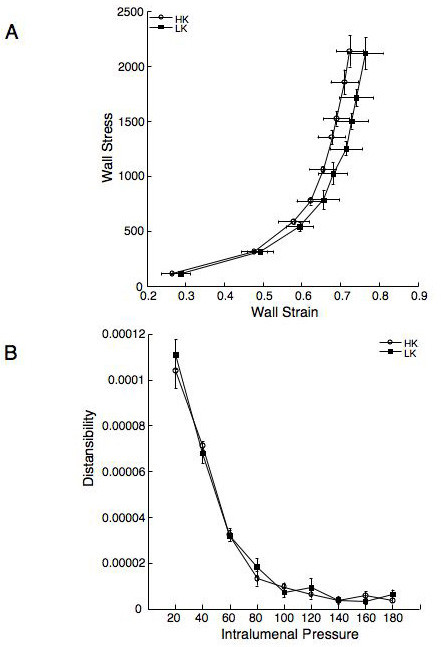
Dietary potassium supplementation has no effect on the vessel compliance as indicated by the stress strain curve (A) or the vessel distensibilty (B). Arteries were mounted in a small vessel arteriograph and vessel structure was assessed as described previously. These results were used to calculate the circumferencial wall stress, wall strain and distensibility. n = 5 for the HK group and 6 for the LK group.

The lumen and outer diameters of the MCA from the rats fed the HK diet were significantly larger over the range of intralumenal pressures than those from rats fed the LK diet (Figure 3A and 3B). Assessment of the vessel wall thickness and the wall to lumen ratio showed there were no significant differences between the rats fed the HK and LK diets (Figure 4A and 4B), suggesting the MCA underwent outward eutrophic remodelling in response to dietary potassium supplementation. Stress strain curves were used to assess the compliance of the MCA, there was no shift in the stress strain curve for the vessels from the rats fed the HK diet compared to the rats fed the LK diet (Figure 5A), similarly, there was no change in the β-coefficient (5.80 ± 1.15 vs 5.97 ± 0.61 HK vs LK). Dietary potassium supplementation also had no effect on the distensibility of the MCA (Figure 5B). The vessel remodelling and growth indices were calculated for the HF rats at an intralumenal pressure of 80 mmHg, the remodelling index was 110.83 indicating outward remodelling in the HK rats and the growth index was -0.41, indicating no growth and a small amount of atrophy.

## Discussion

There are two novel findings in this study. First, we have shown that dietary potassium supplementation reduces the size of the ischemic cerebral infarct in normotensive rats. We have also shown that the MCAs from the rats fed the HK diet have larger lumen and outer diameters. Several studies have shown that reducing the lumen and outer diameters of the MCA reduces the vessels ability to autoregulate [13], and dilate [14], both of these effects may contribute to an increase in the amount of damage caused by cerebral ischemia. Therefore, by increasing the vessel diameters it is possible that the HK diet improved cerebral vessel function and that this is responsible for the reduction in the cerebral infarct size. To the best of our knowledge this is the first study to show a beneficial effect of dietary potassium supplementation in a normotensive rat.

In these studies the rats were fed the specialized diets for six weeks beginning at six weeks of age, this time frame was chosen to mimic the treatment regime used in our previous studies of the effects of dietary potassium supplementation in SHRSP [8]. In that study we observed that dietary potassium supplementation reduced the ischemic cerebral infarct size and improved cerebral vessel structure in hypertensive rats. The results obtained in the current study suggest that potassium supplementation has a similar effect in normotensive rats. Interestingly, the pattern of changes in vessel structure is not the same in the SHRSP and WKY rats. In the SHRSP we observed no change in the lumen diameter of the MCA but reduction in the wall thickness and wall/lumen ratio [8]. This suggests that in the hypertensive rats the HK diet is preventing vessel hypertrophy or hyperplasia. This was not the case in the WKY rats, here the changes in vessel structure with dietary potassium supplementation appear to reflect an outward euthrophic remodeling process, or a increase in the vessel size without a change in the wall thickness, this is supported by the calculations of the remodeling and growth indices. Why the hypertensive rats and normotensive rats respond differently to the HK diet remains unclear. It is possible that the difference in blood pressure between the two strains is responsible for the different responses. Also, in the SHRSP the HK diet caused a significant increase in the plasma potassium concentrations, while there was a trend towards an increase in the WKY rats it did not reach statistical significance. This raises the possibility that there are differences in the potassium handling between the two strains and that may be responsible for the difference in the effect.

The mechanism for the effect of the HK diet on vascular structure in the WKY rats remains unclear. Our previous studies using SHRSP showed that the mRNA levels of various growth factor receptors and collagens were reduced by the HK diet [8]. Because we do not observe a change in vessel wall thickness in the WKY rats it seems unlikely that the HK diet is preventing growth factor stimulated smooth muscle hypertrophy in the WKY rats. Similarly we observed a reduction in vessel compliance and a reduction in collagen mRNA levels in the SHRSP fed the HK diet [8]. Other studies have also suggested that dietary potassium supplementation improves vessel compliance in patients with essential hypertension [15]. In the current study we did not observe a difference in compliance or vessel stiffness, therefore it is unlikely that this is the mechanism for the protective effect of potassium.

It should be noted that the effects of dietary potassium supplementation on the outcome of cerebral ischemia may not be due solely to the changes in lumen diameter. A small increase in potassium concentration causes a reduction in reactive oxygen species (ROS) generation [16]. ROS generation has also been implicated in the pathogenesis of acute ischemic stroke [17], therefore a reduction in ROS generation after the induction of ischemia could be responsible for the beneficial effects of potassium on the outcome of ischemia. Similarly, the pro-inflammatory cytokine, interleukin 1 beta (IL-1β) levels increase rapidly in the brain in response to an ischemic insult [18]. Interestingly, we have shown that dietary potassium supplementation in SHRSP causes a dramatic reduction in the plasma concentration of IL-1β [19], presenting the possibility that this too has an effect on the outcome of ischemia in the normotensive rat.

## Conclusion

In conclusion, we have shown very clear evidence that dietary potassium supplementation reduces the amount of damage caused by cerebral ischemia and increases the MCA lumen and outer diameter in WKY rats. To the best of our knowledge this is the first study to assess the effectiveness of this very simple treatment and cost effective treatment in a normotensive rat.

## Competing interests

The author(s) declare that they have no competing interests.

## Authors' contributions

CSR performed the vessel structure studies, DMP measured blood pressure by telemetry and AMD conceived of the studies, carried out the analysis of cerebral infarct size and prepared the manuscript.
